# People with diabetes adherence to drug, dietary, and lifestyle changes in Erbil city, Iraq

**DOI:** 10.1186/s12902-022-01230-0

**Published:** 2022-12-07

**Authors:** Abubakir Majeed Saleh

**Affiliations:** 1 Department of Community Medicine, College of Medicine, Hawler Medical University, Erbil, Iraq; 2 Department of Nursing, Faculty of Nursing, Tishk International University, Erbil, Iraq

**Keywords:** Diabetes Mellitus, Drug adherence, Iraq

## Abstract

**Background:**

Since diabetes has serious complications that might result in life-long handicaps or even death, it is vital to ensure that people have reasonable control of the disease, which is eventually by good adherence to drugs, diet, and a good lifestyle. People non-adherence to any part of the therapy program for diabetes might result in worsening the condition. This study aimed to evaluate the compliance of people with diabetes to drug, diet, and lifestyle changes in Erbil city, Iraq.

**Patients and methods:**

A descriptive cross-sectional study was conducted among a sample of 288 people with diabetes visiting Layla Qasim Health Center for people with diabetes in Erbil city, Iraq. Data were collected by interviewing the participants and filling out a questionnaire. The survey demonstrated the socio-demographic status, history, information about the participant's condition, frequency of self-monitoring, medication use, the impact of the surroundings and people's concerns, diet, and lifestyle of the participants.

**Results:**

Of 202 participants responded to the questionnaire, 56.9% were female. The mean age was 52.53 ± 13.882 years. 85.6% of the participants were taking the medication regularly, and 78.8% of the participants followed a recommended diet by their doctors. Only 56.4% were exercising, with a majority being male, 70.1%.A strong association was found between gender and doing exercise, educational level and taking the medication regularly, duration of the disease, and following the recommended diet.

**Conclusions:**

The adherence to taking the medication regularly is high, in which single most important cause is following up with their doctors. In contrast, adherence to lifestyle recommendations was suboptimal and essential in managing diabetes. Another reason is that educational level plays a role in understanding the importance of following the recommended lifestyle by the doctor.

## Introduction

Diabetes mellitus (DM) is a condition characterized by the inability of body cells to take up glucose for energy. This results in increased serum glucose levels, known as hyperglycemia. Diabetes that is poorly controlled can have serious complications. It is associated with a potential loss of vision (retinopathy), possible renal failure (nephropathy), and peripheral neuropathy [1].

Diabetes has increased rapidly since the twenty-first century, driven by risk factors such as obesity and a sedentary lifestyle. The impact of diabetes has overwhelmed healthcare systems worldwide, especially in developing countries. Preventing the consequences of diabetes should be a standard, primary goal. Reversing the rising rates of diabetes is an essential step in ensuring full life expectancy [2].

In 2019, diabetes was the ninth leading cause of death worldwide, with an estimated 1.5 million deaths. In 2019, nearly 463 million adults were living with DM rising to (578 million) by 2030 and (700 million) by 2045 [3, 4].

Improper medication adherence is a major cause leading to poor glycemic control among people with diabetes, which worsens the condition and puts people at an increased risk for severe complications, as mentioned earlier. Lack of adherence to drugs increases the rates of hospitalization and mortality. Medication adherence is defined by the world health organization (WHO) as the extent to which a person follows the instructions and medications given by the health care provider. Failure to take medications as prescribed or using less than 80% of the prescribed treatment is termed non-adherence. In people with diabetes, low adherence to anti-diabetic drugs accounts for about 30% to 50% of treatment failure and subsequent complications resulting from rising glucose levels [5].

Various factors significantly contribute to poor drug adherence among people with diabetes. These factors include age, sex, race/ethnicity, education, and income or socioeconomic status. Although non-adherence is a global issue, it is far worse in developing countries with low literacy levels, economic instability, and poor health system [6].

There are multiple types of non-adherence. The first is termed primary non-adherence, in which people are prescribed medications, but the drug is never initiated. Non-persistence adherence is one in which people suddenly stop taking medication. This is usually unintentional, with miscommunication between the people and the provider being the primary cause. The third type of non-adherence is known as non-conforming. This type occurs when medications are not taken as prescribed. This behavior includes skipping doses, taking medicines incorrectly, or taking more than prescribed. The consequence of non-adherence is disease progression, waste of money and medication, and increased hospital visit [7].

Studies have shown the importance of diet and lifestyle changes in managing diabetes. Adherence to diet has also been shown to improve glucose levels, decrease blood pressure, and correct lipid abnormalities, which are factors associated with the micro and macro-vascular complications of diabetes. Some people explain their non-adherence to dietary recommendations based on others' thoughts, poor commitment, and lack of support from their surrounding [8].This study aimed to analyze how people adhere to their medications, diet, and lifestyle and identify the factors causing non-adherence to medication, diet, and lifestyle.

## Patients and methods

### Study design

A facility-based descriptive cross-sectional study was conducted using direct interviews with a sample of people with diabetes in Erbil city, Iraq.

### Study setting

This study was done at Layla Qasim Health Center. Layla Qasim is a specialized center for people with diabetes in Erbil city, Iraq. People with diabetes visit the center for diagnosis, follow-up, and medication refills. Erbil city is the capital of Kurdistan region of Iraq. It is located in the north of Iraq with around 2 Million population.

### Sample size and sampling method

A sample size of 288 people with diabetes was estimated through Epi info software (population size 999999, expected frequency 25%, acceptable margin of error 5%, design effect 1, cluster 1). The required sample size was recruited through a convenient sampling method, and the samples had different socioeconomic backgrounds. The inclusion criteria for the study were being diagnosed with diabetes.

### Time of the study

The study was conducted during a period starting from September to December 2021.

### Data collection

Data were collected through a questionnaire designed by the author based on literature review about this topic and all questions were based on literature review with some modifications to fit the culture of the Kurdistan region, Iraq.The questionnaire included 27 questions distributed in 7 parts. It covered the following information: Socio-demographic status, history and information about the patient's condition, frequency of self-monitoring, medication use, the impact of the surroundings and people's concerns, diet, and lifestyle. The data collection was done through interviews, allowing us to explain all questions to the respondents, especially illiterate people or people with low education levels.

### Data analysis

Data analysis was performed using SPSS for windows (Statistical Package for Social Sciences) version 26. Categorical variables are presented as frequencies and percentages. Numerical variables are reported as the mean ± standard deviation. A chi-square and fisher's exact test were used to demonstrate the association between different variables. A *P*-value of (< 0.05) was considered to be statistically significant.

## Results

The total number of people invited to participate in the study was 288, but 202 participants responded to the questionnaire. The response rate = 70%.

Out of 202 participants, 56.9% were females. The patient's age ranged from 5 to 80 years (mean 52.53 ± 13.882). Those in the age group (51 – 60) represented 37.1% of the participants. 69.8% of the participants were from urban areas. Most of the participants were married, with a percentage of 84.2%. 33.7% of the participants were illiterate. 55% of the patient's BMI was between25 – 29.9 (mean 28.84 ± 13.19). 81.7% of the females were housewives (Table 1).

**Table 1:** Socio-demographic characteristics of the sample

Variable	Frequency	Percentage (%)
**Age (years)**		
1 – 10	2	(1.0)
11 – 20	7	(3.5)
21 – 30	14	(6.9)
31 – 40	7	(3.5)
41 – 50	40	(19.8)
51 – 60	75	(37.1)
61 – 70	46	(22.8)
71 – 80	11	(5.4)
**Gender**		
Male	87	(43.1)
Female	115	(56.9)
**Residency**		
Rural	34	(16.8)
Urban	141	(69.8)
Suburban	27	(13.4)
**Marital status**		
Single	21	(10.4)
Married	170	(84.2)
Widowed	11	(5.4)
**Educational level**		
Illiterate	68	(33.7)
Primary school	48	(23.8)
Intermediate school	29	(14.4)
High school	14	(7.0)
College &postgraduate	42	(20.8)
**Computed BMI**		
18 and less	1	(0.5)
18.5 – 24.9	43	(21.2)
25 – 29.9	111	(55)
30 and more	47	(23.2)
**Occupation**		
Retired	20	(9.9)
Government employed	35	(17.3)
Self-employed	42	(20.8)
Housewife	94	(46.5)
Student	11	(5.4)
Total	202	(100.0)

**Fig. 1:**
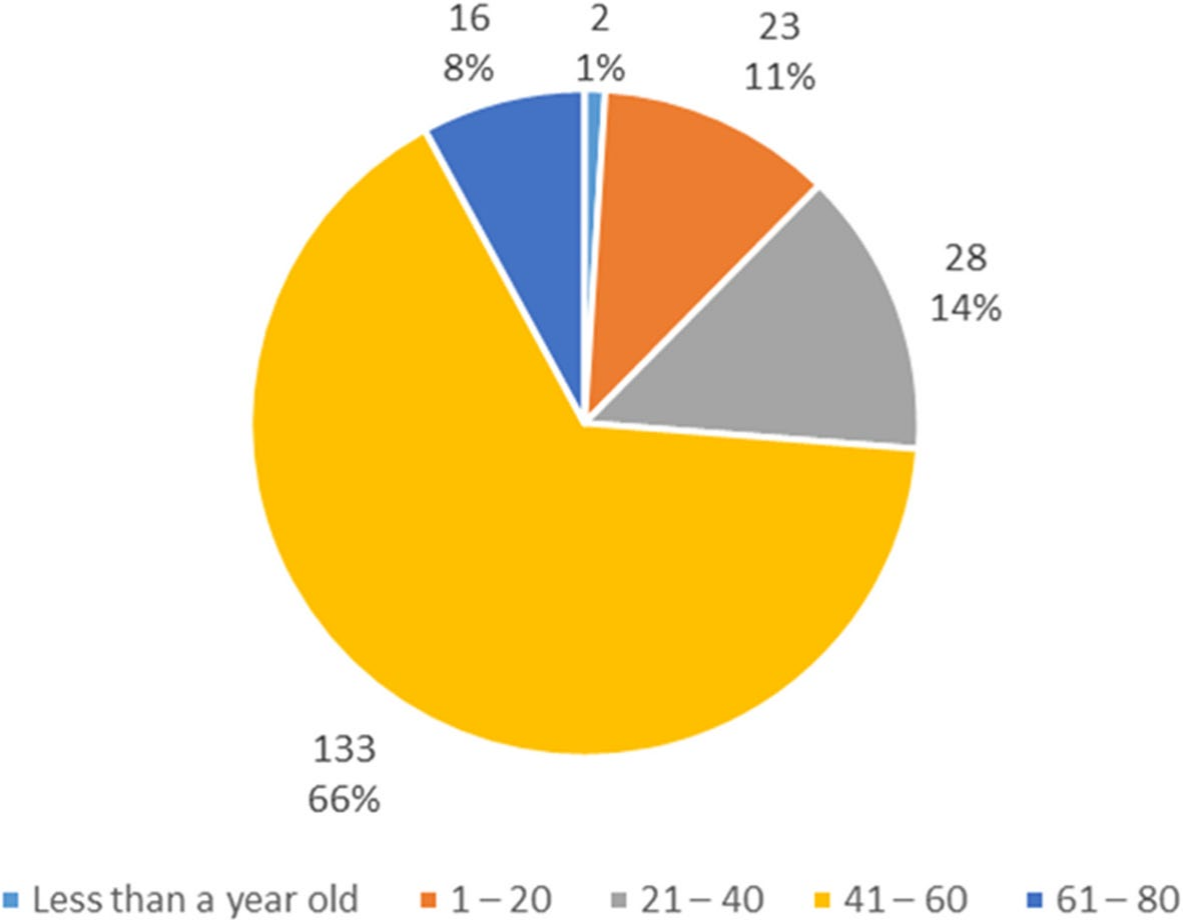
Age at the onset of the disease of the sample

In this study, the onset of the disease was more common in the range (of 41 – 60) years by a percentage of 65.8% (Fig. 1).

**Fig. 2:**
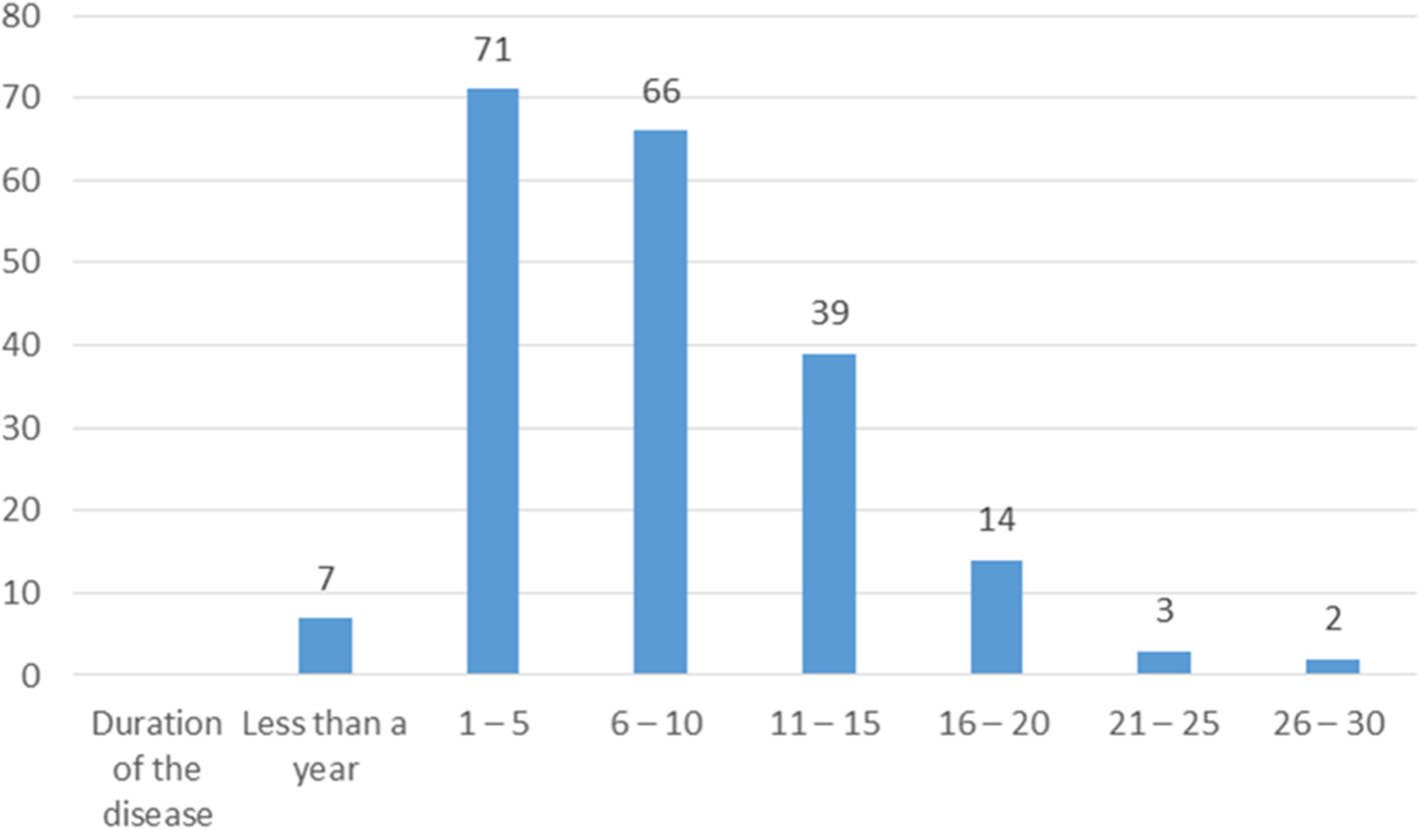
Duration of the disease among the participants

Most participants had the disease for some years, between (1 – 5) years (Fig. 2).

Out of the 202 participants, 85.6% took the medication regularly, and 78.8% followed the diet their doctors recommended. At the same time, the percentage of participants who were doing exercise was 56.4% (Table 2).

**Table 2:** Frequency and percentage of taking the medication regularly, following the diet, and doing exercise

Variable	Frequency	Percentage (%)
**Taking medication regularly**		
Yes	173	(85.6)
No	29	(14.4)
**Following the recommended diet**		
Yes	159	(78.8)
No	43	(21.3)
**Doing exercise**		
Yes	114	(56.4)
No	88	(43.6)
Total	202	(100.0)

In this study, 87.8% of females took their medication regularly. People with intermediate school educational levels had the highest percentage of taking their medication regularly, at 89.7%0.90.7% of participants visiting their physicians more than four times a year took their medication regularly. Participants with BMI (18.5 – 24.9) took their medication regularly by 93.2%.Regarding the duration of the disease; it was found that those participants that had a period of their illness between (16 – 20) years were taking their medication regularly by a percentage of 92.9%. (Table 3).

**Table 3:** Relation of Gender, Educational level, Follow-up, BMI, and duration of disease to Taking medications regularly

Taking medications regularly							
	**Yes**		**No**		**Total**		**P**
	**No**	**%**	**No**	**%**	**No**	**%**	
**Gender**							
Male	72	(82.8)	15	(17.2)	87	(100.0)	
Female	101	(87.8)	14	(12.2)	115	(100.0)	0.319
**Educational Level**							
Illiterate	60	(88.2)	8	(11.8)	68	(100.0)	
Primary School	40	(83.3)	8	(16.7)	48	(100.0)	
Intermediate School	26	(89.7)	3	(10.3)	29	(100.0)	0.11
High school	12	(85.7)	2	(14.3)	14	(100.0)	
College& postgraduate	34	(80.9)	8	(19.1)	42	(100.0)	
**Visitingphysician for follow-up**							
Less than four times a year	43	(72.9)	16	(27.1)	59	(100.0)	
Four times a year	52	(91.2)	5	(8.8)	57	(100.0)	0.004
More than four times a year	78	(90.7)	8	(9.3)	86	(100.0)	
**BMI**							
18 and less	1	(100.0)	0	(0.0)	1	(100.0)	
18.5 – 24.9	41	(93.2)	3	(6.8)	44	(100.0)	0.379
25 – 29.9	93	(83.8)	18	(16.2)	111	(100.0)	
30 and more	38	(82.6)	8	(17.4)	46	(100.0)	
**Duration**							
Less than a year	3	(42.9)	4	(57.1)	7	(100.0)	
1 – 5 years	59	(83.1)	12	(16.9)	71	(100.0)	
6 – 10 years	61	(92.4)	5	(7.6)	66	(100.0)	
11 – 15 years	33	(84.6)	6	(15.4)	39	(100.0)	0.032
16 – 20 years	13	(92.9)	1	(7.1)	14	(100.0)	
21 – 25 years	2	(66.7)	1	(33.3)	3	(100.0)	
26 – 30 years	2	(100.0)	0	(0.0)	2	(100.0)	

Regarding following a recommended diet, 81.6% of the males followed a recommended diet. Those participants who completed college followed a recommended diet by 94.6%. Regarding the role of visiting their physicians,84.9% of those participants that were seeing their doctors for follow-up more than four times a year were following a recommended diet, and 86.4% of the participants with BMI in the range (18.5 – 24.9) were following the recommended diet, 84.6% of those participants that the duration of their disease was some when between (11 – 15) years were following a recommended diet (Table 4).

**Table 4:** Relation of Gender, Educational level, Follow-up, BMI, and duration of the disease tofollowing the recommended diet

Following the recommended diet							
	**Yes**		**No**		**Total**		**P**
	**No**	**%**	**No**	**%**	**No**	**%**	
**Gender**							
Male	71	(81.6)	16	(18.4)	87	(100.0)	
Female	86	(74.8)	29	(25.2)	115	(100.0)	0.306
**Educational Level**							
Illiterate	54	(78.3)	15	(21.7)	69	(100.0)	
Primary School	37	(77.1)	11	(22.9)	48	(100.0)	
Intermediate School	19	(65.5)	10	(34.5)	29	(100.0)	0.006
High school	9	(64.3)	5	(35.7)	14	(100.0)	
College&postgraduate	38	(90.5)	4	(9.5)	42	(100.0)	
**Visitingphysician for follow-up**							
Less than four times a year	40	(67.8)	19	(32.2)	59	(100.0)	
Four times a year	44	(77.2)	13	(15.1)	57	(100.0)	0.053
More than four times a year	73	(84.9)	13	(15.1)	86	(100.0)	
**BMI**							
18 and less	0	(0.0)	1	(100.0)	1	(100.0)	
18.5 – 24.9	38	(86.4)	6	(13.6)	44	(100.0)	0.115
25 – 29.9	83	(74.8)	28	(25.2)	111	(100.0)	
30 and more	36	(78.3)	10	(21.7)	46	(100.0)	
**Duration**							
Less than a year	3	(42.9)	4	(57.1)	7	(100.0)	
1 – 5 years	55	(77.5)	16	(22.5)	71	(100.0)	
6 – 10 years	50	(75.8)	16	(24.2)	66	(100.0)	
11 – 15 years	33	(84.6)	6	(15.4)	39	(100.0)	0.032
16 – 20 years	11	(78.6)	3	(21.4)	14	(100.0)	
21 – 25 years	3	(100.0)	0	(0.0)	3	(100.0)	
26 – 30 years	2	(100.0)	0	(0.0)	2	(100.0)	

Regarding doing exercise, the majority of the males were doing exercise (70.1%).The participants with a college degree were doing exercise by 78.4%0.55.8% of the participants that were seeing their doctors for follow-up more than four times a year were exercising, and 62.2% of participants whose BMI was in the range of (25 – 29.9) were exercising. Participants with a disease duration of less than a year were doing exercise by 85.7%(Table 5).

**Table 5:** Relation of Gender, Educational level, Follow-up, BMI, and duration of the disease to doing exercise

Doing exercise							
	**Yes**		**No**		**Total**		**P**
	**No**	**%**	**No**	**%**	**No**	**%**	
**Gender**							
Male	61	(70.1)	26	(29.9)	87	(100.0)	
Female	53	(46.1)	62	(53.9)	115	(100.0)	0.001
**Educational Level**							
Illiterate	29	(42.0)	40	(58.0)	69	(100.0)	
Primary School	30	(62.5)	18	(37.5)	48	(100.0)	
Intermediate School	17	(58.6)	12	(41.4)	29	(100.0)	0.001
High school	6	(42.9)	8	(57.1)	14	(100.0)	
College& postgraduate	32	(76.2)	10	(23.8)	42	(100.0)	
**Visiting physician for follow-up**							
Less than four times a year	31	(52.5)	28	(47.5)	59	(100.0)	
Four times a year	35	(61.4)	22	(38.6)	57	(100.0)	0.643
More than four times a year	48	(55.8)	38	(44.2)	86	(100.0)	
**BMI**							
18 and less	1	(100.0)	0	(0.0)	1	(100.0)	
18.5 – 24.9	26	(59.1)	18	(40.9)	44	(100.0)	0.032
25 – 29.9	69	(62.2)	42	(37.8)	111	(100.0)	
30 and more	18	(39.1)	28	(60.9)	46	(100.0)	
**Duration**							
Less than a year	6	(85.7)	1	(14.3)	7	(100.0)	
1 – 5 years	45	(63.4)	26	(36.6)	71	(100.0)	
6 – 10 years	39	(59.1)	27	(40.9)	66	(100.0)	
11 – 15 years	13	(33.3)	26	(66.7)	39	(100.0)	0.017
16 – 20 years	7	(50.0)	7	(50.0)	14	(100.0)	
21 – 25 years	2	(66.7)	1	(33.3)	3	(100.0)	
26 – 30 years	2	(100.0)	0	(0.0)	2	(100.0)	

## Discussion

The various complications arising in diabetic patients make it vital to ensure adequate glycemic control of the patients to reduce associated morbidity and mortality. Reasonable control of hyperglycemia in diabetics may be by anti-diabetic medication(s), lifestyle changes, diet, or a combination of all. Adherence to therapies is a primary determinant of treatment success. Failure to adhere is a serious problem that affects the people and the health care system, as concluded by Kao et al [9].

In this study, 85.6% of participants were taking their medications regularly, which are higher than the findings of three previous studies conducted in Iraq [10], Egypt [11], and Saudi Arabia [12], which revealed that 65.4%, 57%, and 67.9% of participants take their medication regularly, respectively. A similar percentage of diabetics’ adherence to therapy was demonstrated in studies conducted in UAE [13] and India [14], which revealed 83.6% and 84%, respectively. This high percentage might be due to the place we took our sample because the majority were people who cared about their health and were following up with their physicians, apart from a minority that was present only for the first time for their diagnosis. Moreover, in a study using direct interviews, the people possibly report only good results that raise the adherence rate.

A study conducted in Iran [15] indicates that adherence to medication protocol reduces the chance of retinopathy, nephropathy, neuropathy, CVD, HTN, and diabetic foot ulcer.

In this study, there was no statistically significant difference between males and females in taking medicines regularly, similar to the findings of the three studies conducted in Iraq [10], UAE [13], and Iran [15], in which there was no gender difference in adherence to medication. Surprisingly, this study found no association between educational level and taking medicines regularly, which contradicts the general belief that more educated people follow therapeutical guidelines more efficiently. Similar findings were achieved in Iran [15] and Nigeria [16]. Physician follow-ups impacted taking medicine regularly; people who visited their physicians four times a year had a higher rate of taking medicines regularly than those who saw their doctors less than four times a year. This concludes that physician follow-ups aid in the overall patient's adherence to medications, diet, and exercise, eventually leading to better glycemic control and decreased complications in diabetes.

This study found an inverse relationship between adherence to medicines and BMI. This finding is similar to the result of another study conducted in Iran [15]. In addition, there was no association between the duration of the disease and taking medicines regularly, as this was also concluded by research done in Iraq [10].

According to Barclay et al. [17], dietary modification is required for type 2 diabetes mellitus patients. Regarding following diet recommendations in our study, 78.7% of the people stated that they were following the diets recommended for them, which was higher than the percentages found in another study conducted in Iraq which was 10.8% [10]. This finding might result from the increased access to guidelines, programs, presentations, and articles about food and diet found on the Internet and Television. It might also be a consequence of the ease of reaching and communicating with dietitians and doctors; another possible explanation for this could be the growing awareness of people with diabetes that has contributed to the majority of people in our sample following the recommended diet.

The effect of gender on the following diet revealed that males followed the diet more than females; However, the difference was not so much it was a bit surprising since, in our culture, females are more often responsible for food preparation. There was an interesting association between following diet and physician follow-up frequency, in which the more patients followed up with their physicians, the more they followed dietary recommendations; again, we see that follow-ups lead to better outcomes in people's performance toward recommendations for their health. We found that 90% of people with a BMI of 18.5–24.9 followed the recommended diet, and the rate was lesser in others. We also found that 84.9% of people who were exercising were following their recommended diet; this might arise from the person’s attitude towards attempts to be in better health.

Lifestyle modifications and exercising are crucial for glycemic control and decreasing diabetes-associated morbidities and mortalities. Adhering to a healthy lifestyle and exercising was associated with a reduced mortality rate of 57% for people with diabetes. Even more significant decreases in the rate of diabetes-associated complications was reported by the study from Schlesinger et al^.^ [18]. Adherence to recommended lifestyle and exercise in this study was 56.4%, which is higher than the results of a study done by Mukherjee et al [19]. However, we found that males tend to exercise more than females in which there were similar findings in a study done in Iraq [10]. There was no significant association between following exercise and lifestyle recommendations and education level. An interesting association between physician follow-ups and following exercise and lifestyle recommendations was found. Those who followed up with their physicians four times a year followed exercise and lifestyle recommendations more efficiently than those who followed up with their physicians less than four times a year. This finding shows the remarkable impact of physicians on people, as following up with the doctor regularly increases the chance of the people's exposure to medical education and recommendations, which results in a better outcome. These findings are supported by a previous study by Di Loreto C et al [20]. In contrast, there was a significant association between BMI and following exercise and lifestyle recommendations in which people with lesser BMI were following exercise and lifestyle recommendations more efficiently, in contrast, a study conducted in Iran showed no relationship between BMI and physical activity in people with diabetes [15].

### Limitations of the study

This study was done through cross-sectional design, which may limit the generalizability of the finding and cannot show the causal relationship, particularly the effect of education level on adherence. The sample size is relatively small, which might not represent the whole population, and makes the difference only judged by the *P*-value. Moreover, it was better to discuss the relationship between different variables using logistic regression.

## Conclusions

The adherence to taking medications regularly is high, in which single most important factor is following up with their physicians. In comparison, adherence to lifestyle recommendations was suboptimal, with males exercising more than females. However, following diet recommendations showed no difference in gender—the patient's educational level plays a role in following the recommended lifestyle changes.

## Data Availability

The datasets used in this study are available with the corresponding author upon reasonable request.
